# Reply: Some considerations on digital health validation

**DOI:** 10.1038/s41746-019-0176-z

**Published:** 2019-10-17

**Authors:** Simon C. Mathews, Michael J. McShea, Casey L. Hanley, Alan Ravitz, Alain B. Labrique, Adam B. Cohen

**Affiliations:** 1 Armstrong Institute for Patient Safety and Quality, 750 E Pratt St, 15th Floor, Baltimore, MD 21202 USA; 2 Johns Hopkins Medicine, Department of Internal Medicine, Division of Gastroenterology, 1800 Orleans St, Baltimore, MD 21287 USA; 3 Health Technologies, National Health Mission Area, The Johns Hopkins University Applied Physics Lab (APL), 11100 Johns Hopkins Rd, Laurel, MD 20723 USA; 4 Johns Hopkins Bloomberg School of Public Health, JHU Global mHealth Initiative, 615N. Wolfe St, Baltimore, MD 21205 USA; 5 The Johns Hopkins Hospital, Department of Neurology, 1800 Orleans St, Baltimore, MD 21287 USA

**Keywords:** Technology, Health policy

We thank van Velthoven and Smith for their thoughtful comments regarding our approach to validating digital health. They introduce several points that could benefit from further clarification and future investigation.

Regarding the issue of scope and setting, we believe our approach is flexible enough to be adapted to address both the range of clinical contexts (e.g. home based and acute care) and the variety of solution types (e.g. mobile apps, wearables, artificial intelligence-based decision support). However, not all domains of evaluation (i.e. clinical, technical, usability, cost) will be as relevant or robustly evaluated depending on the technology capability and the context of the use. The larger question of how to determine which solutions should be evaluated remains open to debate and will likely be a function of who is able to support this type of evaluation—essentially the broader the support, the larger the scope.

Given the current fragmentation in healthcare, a stakeholder-centric approach may provide a practical guide. For example, patient advocacy groups could support evaluation in technologies most relevant or used by their constituency. Physician specialty societies may select emerging technologies relevant to their field. Payors could focus on technologies that they believe have the greatest impact on minimizing harm and costs. Industry participants could seek to differentiate their products by undergoing more rigorous evaluation. Relying on existing digital gatekeepers such as app stores to maintain quality standards as they relate to health content is likely an unrealistic expectation as it would require greater investment and expertise on their part and is at odds with the goal of increasing the variety and availability of products in their marketplaces.

van Velthoven and Smith highlighted that there may be little need for extensive product evaluation since the majority of solutions do not appear to cause significant harm. We believe this is, in part, due to the lack of systematic evaluation to assess product safety and to the relatively low technical capabilities of the technologies that are most widely accessible. To this latter point, as diagnostic sensors and therapeutic components advance, the potential for greater harm will also increase. Even in the current relatively “low tech” environment, however, harm may be occurring, albeit covertly, and in ways that are intangible and difficult to measure. In this context, the historical comparison to health information websites is apt. Clinic patients commonly inquire about medical information read online, which can be a frequent source of misunderstanding and anxiety. While unlikely to be causing physical harm, this misinformation likely has clinical impact. Analogously, even well intentioned “low tech” apps can generate confusion and potentially misguided or incorrect recommendations, which can adversely affect health. For example, we have discovered in our pilot that focuses on oncology apps assessment, some information is inaccurate or patently false. With respect to overall standards in digital health, the National Institute for Health and Care Excellence teamed with the National Health Service published a promising framework (concurrent with the release of this work) for different levels of evidence that is robust.^1^

Regarding the need for incorporating end user and subject matter expertise early in the product lifecycle, we agree and believe this input should be solicited or integrated within every step including identifying “needs” as suggested by van Velthoven and Smith. In our pilot we have made extensive use of user centered design (UCD) principles to define requirements against which the utility of the candidate apps is evaluated. Both patient and clinician viewpoints have been incorporated in the UCD process to ensure these perspectives are adequately represented in the evaluation.

We also agree it is important to recognize the challenges that other multi-stakeholder efforts have encountered. Additionally, one should incorporate experience from low and middle income countries since uptake of digital tools may be faster and in far greater numbers, given limitations in traditional health infrastructure and resources. Finally, we also agree that achieving the multi-stakeholder vision proposed is challenging and will benefit from tangible demonstration of value which we are currently undertaking. The need for a transparent, comprehensive, and standards-based assessment that is guided by end-user requirements is clear and will only continue to grow. We believe our approach provides one feasible path that provides both structure and flexibility.
